# Outcomes of thrombolytic therapy in prosthetic valve thrombosis; a single centre study

**DOI:** 10.12669/pjms.42.9.13490

**Published:** 2026-09

**Authors:** Ikram Ahmed Rana, Fatina Munawar, Ayesha Mubasher, Muhammad Talha Bajwa

**Affiliations:** 1 Ikram Ahmed Rana, MBBS FCPS. Tahir Heart Institute, Fazl-e-Omar Hospital, Chenab Nagar, District Chiniot, Pakistan; 2 Fatina Munawar, MBBS. Tahir Heart Institute, Fazl-e-Omar Hospital, Chenab Nagar, District Chiniot, Pakistan; 3 Ayesha Mubasher, MBBS, MD. Tahir Heart Institute, Fazl-e-Omar Hospital, Chenab Nagar, District Chiniot, Pakistan; 4 Muhammad Talha Bajwa, MBBS, MD.Services Hospital Lahore, Lahore, Pakistan

**Keywords:** Thrombosis, Thrombolytic, Valve, Prosthetic

## Abstract

**Objective:**

To determine the outcomes of thrombolytic therapy in prosthetic valve thrombosis.

**Methodology:**

Data was taken from the hospital discharge database, of the Tahir Heart Institute, for patients admitted from January 01, 2016 to June 30, 2024. Definitive diagnosis was made with transthoracic echocardiography showing an obstructed prosthetic valve for patients presenting with new-onset dyspnea. Streptokinase infusion was given per ACC/AHA guidelines with twelve-hourly TTE for transvalvular gradient assessment. The primary outcome was successful thrombolysis with reduction in transvalvular gradients. Data was analyzed by Microsoft Excel. Patients were followed up over time.

**Results:**

Approximately 0.1% of total hospital admissions, i.e. twenty-seven cases, were found to have prosthetic valve thrombosis. Target gradients on TTE were achieved for 71% (n=17) of mitral and 100% (n=3) of aortic valves: thrombolysis was successful in 74% (n=20), partial success in 18.5% (n=5) and unsuccessful in 7.4% (n=2) of cases. Subsequent complications were: myocardial infarction 7.4% (n=2), embolic phenomena 14.8 % (n=4), coronary emboli 7.4% (n=2), brain emboli 7.4% (n=2), re-thromboses 7.4% (n=2) and in-hospital mortality 7.4% (n=2) of cases. The mean time for follow-up after discharge was 3.04 ± 2.07 years; one patient died a year after discharge.

**Conclusion:**

Thrombolytic therapy with streptokinase has been shown to be an effective and relatively safe method for managing prosthetic valve thrombosis.

## INTRODUCTION

Mechanical prosthetic valve thrombosis (PVT) is an unusual yet life-threatening condition, requiring prompt diagnosis and treatment.^1^ Some factors may cause mechanical heart valves to malfunction: thrombosis, pannus ingrowth, degeneration, and endocarditis.^2^ The patients can present with a variable picture, ranging from mild shortness of breath to severe hypoxic respiratory failure. Hence, an acute onset of symptoms in an individual having a prosthetic valve (PV) necessitates timely assessment and management.^3^

As per the 2020 ACC/AHA Guideline for the management of patients with valvular heart disease, thrombosis of left-sided mechanical valves should be managed immediately, with either fibrinolytic therapy or surgical intervention, based on several considerations.^4^ The location of the valve, its obstruction, and presentation may help determine the strategy.^5^ According to the 2021 ESC/EACTS guidelines for valvular heart disease, urgent surgical intervention is indicated for obstructive valve thrombosis unless there are coexisting conditions that may preclude surgery. Other strategies include intensive anticoagulation and management with thrombolytic therapy (TT).^6^ There has been an increase in choosing TT over surgery for mechanical PVT in the last ten years.^7^ Moreover, urgent TT is more feasible than immediate surgery in developing countries owing to several factors such as unavailability of personnel and equipment for cardiothoracic surgeries for emergency cases, financial constraints, and apprehension regarding open-heart surgeries. On the other hand, the accessibility of streptokinase with its administration by the less experienced physicians for acute care makes it a more favorable approach towards management of PVT. Considering the architecture of healthcare in resource-limited countries, this study presents the outcomes of TT for twenty-seven cases that were managed in one center, in terms of reduction in transvalvular gradient on TTE, to suggest that the TT is an effective and somewhat safe management strategy.

## METHODOLOGY

In this observational study, patients presenting to the emergency department, from January 1, 2016, to June 30, 2024, who had PV and a new-onset shortness of breath, an absence of a mechanical click on precordial examination, and confirmation of obstructed PV on TTE, were enrolled. The data were collected retrospectively from the electronic discharge database of the hospital, Tahir Heart Institute, a tertiary care hospital, located in a suburban area with limited resources. The patients were followed up post-treatment, and their clinical profiles were noted for the long-term outcomes of TT over the same period: January 2016 to June 2024. The number of hospital admissions during this period was 26081; it was found out that only twenty-seven cases had PVT: approximately 0.1 percent of hospital admissions.

### Inclusion & Exclusion Criteria:

The enrolled cases were selected based on the inclusion criteria: all male and female patients, who had single or double valve replacements, and also the patients who had combination of valve replacement with coronary artery bypass grafting. The patients who were excluded: either had hypersensitivity to SK, or SK infusion within the last six months, or asymptomatic non-obstructive valve thrombosis, or non-thrombotic increase in transvalvular gradient on transthoracic echocardiography.

### Ethical Approval:

The study has been approved by the Institutional Review Board committee of the Tahir Heart Institute (Reference# IRB/THI/001/25); date: January 4, 2025.

Patients were provided information regarding TT and redo surgery. For those who opted for TT: informed consent was taken; SK infusion was given 250,000 units in the first half hour, followed by 100,000 units per hour. TTE was repeated every 12 hours. Mean pressure gradients were assessed on TTE. If significant improvement (transvalvular gradients close to normal) was noted in pressure gradients, then SK infusion was stopped and patients were started on Heparin and Warfarin and dosages were titrated until an acceptable INR was achieved. Otherwise, SK infusion was continued for a maximum of 48 hours.

Baseline characteristics such as time since the surgery, type of valve (mechanical or bioprosthetic), single or double valve replacement, medication compliance, INR, and mean trans-valvular gradient on echocardiogram before SK infusion were noted.

Successful thrombolysis was defined as a trans-valvular gradient less than the mid value of moderate severity range for that valve. It was 33 mmHg for aortic valve (AV) (Moderate range 25 to 40) and 7mmHg (Moderate range 5 to 10) for mitral valve (MV). A post-SK transvalvular gradient of 33 mmHg and 7mmHg or more but less than baseline value (value at presentation) for AV and MV, respectively, was considered partial success. While it was considered unsuccessful if no reduction in trans-valvular gradient was noted. In-hospital mortality and complications like major bleeding, cerebrovascular accidents, myocardial infarction, embolic events, and re-thromboses were noted.

### Statistical Analysis:

Data was analyzed by Microsoft Excel. Quantitative variables were summarized by mean and standard deviation. Qualitative variables were summarized by frequency and percentage.

## RESULTS

A total of 27 cases had PVT. There were more male patients (n=17, 63%) than female patients (n=10, 37%), their mean age was 38 ± 10.5 years. Twenty (74%) patients had mitral valve replacement (MVR), six (22%) patients had dual valve replacement (DVR), i.e., MV and AV, and only one (4%) patient had aortic valve replacement (AVR). The majority of patients had subtherapeutic INR (n=21, 78%). INR within the reference range was seen in four patients (n=4, 15%), while two patients (n=2, 1%) had supra-therapeutic INR. The mean time of the presentation with thrombosis since the procedure was 3.1 ± 4.06 years. Only two patients had bioprosthetic valve replacement. The baseline characteristics of the patients are presented in Table-I. The outcomes of SK infusion in terms of reduction in trans-valvular gradient and complications are presented in Table-II and Table-III, respectively. The target gradient on TTE was achieved in 71% (n=17) of MV cases and 100% (n=3) of AV cases. Compiled data of both valves showed successful thrombolysis: 74% (n=20). Partial success was achieved in 18.5% (n=5) of the cases, and it was unsuccessful in 7.4% (n=2) of the cases.

**Table-I:** Baseline characteristics.

Baseline characteristics	
Age in years (Mean ± SD)	38.15 ± 10.5
Follow up time in years (Mean ± SD)	3.04 ± 2.07
Time since procedure in years (Mean ± SD)	3.1 ± 4.06
Female n (%)	10 (37)
Male n (%)	17 (63)
Subtherapeutic INR n (%)	21 (78)
MVR n (%)	20 (74)
DVR n (%)	6 (22)
AVR n (%)	1 (4)
Supratherapeutic INR n (%)	2 (7)
Therapeutic-range INR n (%)	4 (14.8)
Bioprosthetic valves n (%)	2 (7)

**Table-II:** Streptokinase success rate.

	n (%)
Successful Thrombolysis	20 (74)
Partially successful thrombolysis	5 (18.5)
Unsuccessful thrombolysis	2 (7.4)

**Table-III:** Complications of thrombolytic therapy.

Complications	n (%)
Acute Myocardial Infarction	2 (7.4)
Rethrombosis	2 (7.4)
Embolic Phenomenon	4 (14.8)
Major Bleed	0 (0)
In-Hospital Mortality	2 (7.4)

Some patients developed complications due to SK infusion, as presented in Table-III. Myocardial Infarction after SK therapy was observed in 7.4% (n=2) of cases.; Thromboembolism in 14.8 % (n=4) of patients, coronary emboli (CE) in two patients, and brain emboli in the other two cases. Re-thromboses were noted in 7.4% (n=2) of cases. There was 7.4% (n=2) in-hospital mortality because of unsuccessful thrombolysis with SK infusion. However, no patient developed major bleeding complications. Patients were followed up over time for delayed complications, the mean time for follow-up was 3.04 ± 2.07 years. One patient died about a year after the PVT episode and SK infusion.

## DISCUSSION

The percentage of new PVT cases reported each year is 0.3-1.3 per total population of patients having prosthetic valves.^1^ The AHA guidelines have proposed equal benefits of surgical intervention and TT for managing PVT.^4^ Contrary to this, EACTS guidelines prioritize surgery as the first-line treatment-option.^6^ Four patients in our study had PVT with INR in the therapeutic range; such concurrence of PVT and therapeutic INR range has been documented by Foley PW, et al., in a case report. Hence, they advise considering PVT as one of the differential diagnoses, unless excluded after thorough clinical workup, in patients who have PV and arrive with tell-tale clinical scenario of pulmonary oedema.^8^

Two patients had supratherapeutic INR: Farzaneh K, et al. investigated the safety profile of TT in patients with PVT who had high INR levels: the range of INR was 0.9 to 4.9, with a median value of 2.1. It was concluded that there was no notable aggravation of the clinical condition in terms of major and minor bleeding complications based on p-values greater than 0.05, and it also became evident that the response to SK infusion was similar at different levels of INR.^9^

In our study, the management of PVT was successful in 74% of patients, which was comparable to a study done in India that included 110 patients, in which 81.8% of patients showed complete response to therapy and 11% of patients showed partial response to therapy; failure in 8.2% of patients.^10^ Abbas S, et al. conducted a study to determine the efficacy of TT for PVT, also based in a tertiary care hospital in Pakistan: they found out that a large proportion of patients presenting with PVT had INR in the subtherapeutic range that resulted due to various reasons such as lack of insight about the significance of maintaining INR in a therapeutic range, seldom shortage of warfarin at pharmacies, a dearth of accessible facilities both by affordability and locality, and randomly becoming non-compliant to medication at times.^11^ The aforementioned study had successful thrombolysis in 66.7% of patients, and it was inferred that patients having NYHA class I/II at presentation had promising results; however, with NYHA class III/IV, the probability of fatal outcomes after SK infusion was higher.^11^

Our study had an overall mortality of seven percent. Another retrospective observational study aimed to assess the significance of follow-up of patients managed with TT for PVT sixty-six patients were enrolled; fifty-nine patients had obstructed MV, and only seven patients had obstructed AV. Sixty-one patients had good outcomes in that the obstruction was resolved; however, five patients had fatal outcomes, similar to our study, i.e., 7.57%. With an average follow-up of 22.7 ± 20.9 months, 68.85% of patients were alive, and five patients were lost to follow-up; 22.95% of patients died. It was inferred that following successful TT, there was an increased probability of mortality within two years of follow-up, and regular follow-ups could help improve prognosis.^12^

A retrospective study consisted of thirty-nine patients who underwent redo-surgical valve replacement for PVT. The factors affecting the results pre-, intra-, and post-operatively were investigated. The majority, i.e., 64.1 percent of patients, had a subtherapeutic INR i.e., less than two; 82.1 percent of patients had mechanical PVT, with 76.9 percent of patients having thrombosis in the MV. The percentage of fatal outcomes in thirty days of the procedure was 7.7, and it was found that a prior attempt to undergo TT significantly increased the possibility of mortality in thirty days of the surgical intervention, in contrast with the direct approach of managing with surgery, with a p-value of 0.018. Hence, it was concluded that surgery for PVT had a good prognosis if no initial attempt was made to manage via TT.^13^

As relapse due to recurrent PVT is one of the undesired complications of partially obstructed PV, two out of the twenty-seven patients had re-thromboses: the patients had post-SK transvalvular gradients of 11 mmHg and 31 mmHg for their respective mitral and aortic prostheses. In a systematic review and meta-analysis that included fifteen studies involving 1235 patients, surgical valve replacement and fibrinolytic therapy were compared. The outcome of re-thrombosis was significantly higher in patients who had fibrinolytic therapy, owing to a risk ratio of 2.06 with a 95% confidence interval (pooled RR = 2.06, 95% CI: 1.29–3.27, I² = 0.0%).^14^ The difference in the occurrence of re-thrombosis can be accredited to this being a single-center study with a small number of patients and cases as compared to the above-mentioned meta-analysis. According to our PubMed-based literature review, only a few studies report data concerning re-thromboses following successful thrombolysis.

Thromboembolism also complicates management with TT, in our study, it occurred in 14.8% of patients. Another single-centre study, enlisted seventy-two patients, with thromboembolic phenomena in only 4.17% of patients.^15^ None of the patients in our study suffered any major bleeding complications, in accordance with the aforementioned study, in which it was found in only one patient.^15^ Acute myocardial infarction (AMI) was considered distinct from CE according to NCVC (National Cerebral and Cardiovascular Center) criteria,^16^ and AMI occurred in only two patients in our study.

Chopard R, et al. conducted a systematic review and meta-analysis to compare the outcomes of TT against surgery for PVT, by including sixteen studies with 1389 patients who had PVT. The patients who had thrombolysis showed a mortality rate of 10.8% whereas the surgical intervention group had a mortality rate of 15.3%. The pooled risk difference post-thrombolysis versus post-surgery was 1.13 (exact confidence interval, 0.74 - 1.79, p<0.001). It was also discovered that the studies that employed low-dose or slow-infusion of the tissue plasminogen activator had lower mortality rate, in comparison with studies that used surgical intervention. The data analysis depicted that TT was a favourable therapeutic strategy for PVT without cardiogenic shock. It was inferred that future observational studies or randomized controlled trials were required to gather conclusive evidence.^17^

Considering the promising results of TT in multiple studies with fewer complications, and the risks associated with surgery, TT should be further brought into practice, and more studies, especially clinical trials, comparing the two therapeutic strategies are required. This can be particularly beneficial for low-income countries where surgical facilities are limited, and mortality and other complications are frequent.

### Limitations:

As it is a single-center study, the findings may not apply to other healthcare settings and the small sample size may limit their generalizability. Further limitations include the “retrospective” collection of data and a lack of a control group, such as a surgical intervention group.

## CONCLUSION

This study demonstrates that TT with streptokinase is effective and relatively safe for managing PVT, particularly in resource-limited settings like Pakistan.

### Authors’ Contribution:

**IAR:** Idea, data collection, data analysis, supervision, critical review,

**FM and AM:** Manuscript writing, editing, revision, accuracy of the manuscript.

**MTB:** Data analysis, compilation of results, revision.

All authors have read and approved the final version and are accountable for the integrity of the study.
